# Whole-Genome Analysis of Genomic Diversity and Antimicrobial Resistance Among Clinical *Klebsiella pneumoniae* Isolates from Farwaniya Hospital in Kuwait

**DOI:** 10.3390/antibiotics15100960

**Published:** 2026-09-28

**Authors:** Fatemah Alatar, Samar S. Boswihi, Hussain A. Safar, Anfal Al-Adwani, Abu Salim Mustafa, Wadha Alfouzan

**Affiliations:** 1 Serology and Molecular Microbiology Reference Laboratory, Mubarak Al-Kabeer Hospital, Ministry of Health, Kuwait City 13110, Kuwaitabu.mustafa@ku.edu.kw (A.S.M.); 2 Dasman Diabetes Institute, Kuwait City 15462, Kuwait; 3 Department of Microbiology, College of Medicine, Kuwait University, Kuwait City 13110, Kuwait; 4 Innomedics Medical Company, Kuwait City 13114, Kuwait; 5 Microbiology Unit, Department of Laboratory Medicine, Farwaniya Hospital, Ministry of Health, Kuwait City 81004, Kuwait

**Keywords:** *Klebsiella pneumoniae*, antibiotic resistance, whole-genome sequencing, wgMLST

## Abstract

**Background:** *K. pneumoniae* is a major opportunistic pathogen recognized by the World Health Organization as a critical-priority antimicrobial-resistant pathogen. This study characterized isolates for antibiotic resistance determinants, virulence-associated genes, mobile genetic elements, and population structure of the isolates, and evaluated the correlation between antimicrobial resistance genotype and phenotype. **Methods:** Ninety-five isolates were collected from different clinical specimens in August–December 2024 in Farwaniya Hospital, Kuwait. Fifty-six isolates were selected for whole-genome sequencing (WGS) using Oxford Nanopore Technology. Antimicrobial resistance genes, virulence-associated genes, plasmid replicons, insertion sequences, transposons, and sequence types were identified using different bioinformatics tools. **Results:** The 56 isolates comprised 47 multidrug-resistant and 9 non-multidrug-resistant isolates. β-lactam resistance genes were detected in all isolates, with *bla*_SHV_ (96.4%), *bla*_CTX-M-15_ (75.0%), *bla*_OXA_ (64.3%), and *bla*_NDM-5_ (30.4%) being the most prevalent. IncFIB(K) was the predominant plasmid replicon, whereas ISSen9, IS26, and IS903 were the most common insertion sequences. Twenty-eight virulence-associated genes were identified, with enterobactin-associated genes, fimbrial adhesion genes, and *ompA* detected in over 90% of the isolates. MLST identified 32 sequence types, including eight novel sequence types, with ST16 and ST147 predominating. High genotype–phenotype concordance was observed for cephalosporins (87.5%) and penicillins (85.7%). **Conclusions:** *K. pneumoniae* isolates exhibited extensive genomic diversity and multidrug resistance associated with multiple resistance determinants, mobile genetic elements, and high-risk lineages. These findings highlight the value of WGS for antimicrobial resistance surveillance and support continued genomic monitoring to support infection prevention and antimicrobial stewardship programs in Kuwait.

## 1. Introduction

*Klebsiella pneumoniae* is an opportunistic Gram-negative pathogen belonging to the family Enterobacteriaceae. It is responsible for a wide range of healthcare-associated and community-acquired infections, including pneumonia, bacteremia, urinary tract infections (UTIs), wound infections, liver abscesses, and septicemia, particularly among immunocompromised and hospitalized patients [1]. *K. pneumoniae* is considered a critical-priority antimicrobial-resistant (AMR) pathogen by the World Health Organization, requiring urgent research and development of new therapeutic strategies [2]. In addition, it is recognized as one of the leading causes of mortality associated with antimicrobial resistance worldwide [3]. The rising antibiotic resistance in *K. pneumoniae* isolates has significantly limited available therapeutic options and contributed to poor clinical outcomes and increased mortality rates [4].

*K. pneumoniae* isolates exhibit resistance to several clinically important antibiotic classes, including β-lactams, aminoglycosides, fluoroquinolones, and tetracyclines [5]. Resistance to β-lactams is primarily mediated by the production of β-lactamases, including extended-spectrum β-lactamases (ESBLs), cephalosporinases (AmpC), and carbapenemases (KPC) [5,6]. The most prevalent ESBL enzyme families among the ESBL-producing isolates include SHV, TEM, OXA, and CTX-M, which are plasmid-borne and capable of hydrolyzing penicillins, expanded-spectrum cephalosporins, and monobactams [5,7]. These enzymes can be inhibited by β-lactamase inhibitors such as clavulanic acid, tazobactam, and sulbactam [7]. The AmpC β-lactamases confer resistance to the 3rd- and 4th-generation cephalosporins and are not effectively inhibited by clavulanic acid but are inhibited by cloxacillin or boronic acid [8].

The emergence of carbapenem-resistant *K. pneumoniae* (CRKP) is of particular concern because carbapenems are considered the last resort for the treatment of multidrug-resistant *K. pneumoniae*. Carbapenem resistance is mediated by carbapenemases belonging to Ambler molecular classes A (e.g., KPC and GES), B (e.g., NDM, VIM, and IMP), and D (e.g., OXA-48-like), which hydrolyze carbapenems such as imipenem, meropenem, and ertapenem [9,10]. In addition to carbapenemase production, resistance may also result from porin loss combined with ESBL or AmpC production, further complicating treatment strategies [11].

Aminoglycoside resistance in *K. pneumoniae* is mostly mediated by plasmid-borne aminoglycoside-modifying enzyme-encoding genes, *aac*, *aph*, and *ant*, which enzymatically modify and inactivate aminoglycosides [12]. Other resistance mechanisms include efflux pump overexpression and methylation of the 16S rRNA target site [12]. Fluoroquinolone resistance is primarily mediated by chromosomal mutations in the quinolone resistance-determining regions (QRDRs) of the *gyrA*, *gyrB*, *parC*, and *parE* genes, which encode DNA gyrase and topoisomerase IV, respectively [12]. In addition, reduced fluoroquinolone susceptibility and development of high-level resistance can be attributed to plasmid-mediated quinolone resistance genes, such as *qnr* genes [12]. Colistin resistance in *K. pneumoniae* isolates has been attributed to chromosomal mutations or acquisition of plasmid-mediated *mcr* genes; however, colistin resistance is less commonly reported compared to other antibiotics [13].

In addition to multidrug resistance, the pathogenicity of *K. pneumoniae* is also due to its virulence factors that promote colonization, persistence, immune evasion, and invasive disease [1]. The polysaccharide capsule is a major virulence factor that protects the bacterium from phagocytosis and complement-mediated killing. The lipopolysaccharide (LPS) O-antigen contributes to serum resistance and immune evasion, while fimbriae and pili facilitate adhesion to host tissues and biofilm formation [1]. Siderophores are small iron-binding metabolites that enable iron acquisition, which is essential for bacterial growth and survival within the host. In addition, outer membrane proteins (OMPs) and efflux pump systems, such as AcrAB-TolC, assist in both antimicrobial resistance and virulence by reducing intracellular antibiotic accumulation and enhancing bacterial survival under stressful conditions [1].

The rapid global dissemination of multidrug-resistant (MDR) *K. pneumoniae* strains highlights the need for continuous epidemiological surveillance and molecular characterization of the circulating MDR strains. Although conventional phenotypic antimicrobial susceptibility testing provides valuable information on the resistance profiles of bacterial isolates, it may not fully elucidate the underlying antibiotic resistance mechanisms. WGS has been considered a powerful and comprehensive tool to identify antimicrobial resistance genes, virulence-associated factors, plasmid replicons, and phylogenetic relationships among bacterial isolates [14]. Correlating the genetic determinants obtained from WGS with the phenotypic susceptibility patterns expands the understanding of resistance mechanisms and supports the development of guidelines for infection prevention and antimicrobial stewardship programs. This study aimed to identify the antimicrobial resistance determinants in *K. pneumoniae* isolates obtained from Farwaniya Hospital, Kuwait, using WGS and to compare the genomic findings with the phenotypic antimicrobial susceptibility patterns.

## 2. Results

### 2.1. Isolate Characteristics and Antimicrobial Susceptibility Profiles

The patients’ demographic data are shown in Table 1. The *K. pneumoniae* isolates were obtained from different clinical samples comprising urine (*n* = 37), blood (*n* = 17), anal swab (*n* = 16), wound swab (*n* = 6), sputum (*n* = 5), endotracheal secretion (*n* = 4), throat swab (*n* = 3), tissue (*n* = 3), rectal swab (*n* = 3), and tracheal aspirate (*n* = 1).

The susceptibility of the 95 *K. pneumoniae* isolates was determined against 33 antibiotics. The isolates were resistant to cefuroxime (*n* = 63; 66.3%), ceftazidime (*n* = 62; 65.2%), ampicillin (*n* = 57; 60.0%), cefepime (*n* = 53; 55.7%), amoxicillin-clavulanic acid (*n* = 45; 47.3%), trimethoprim/sulfamethoxazole (*n* = 44; 46.3%), piperacillin/tazobactam (*n* = 42; 44.2%), ciprofloxacin (*n* = 41; 43.1%), cefotaxime (*n* = 39; 41.0%), nitrofurantoin (*n* = 34; 35.7%), ampicillin-sulbactam (*n* = 34; 35.7%), cefazolin (*n* = 33; 34.7%), ceftriaxone (*n* = 31; 32.6%), meropenem (*n* = 32; 33.6%), imipenem (*n* = 28; 29.4%), ertapenem (*n* = 25; 26.3%), gentamicin (*n* = 24; 25.2%), aztreonam (*n* = 22; 23.1%), levofloxacin (*n* = 21; 22.1%), cefoxitin (*n* = 16; 16.8%), cefuroxime-axetil (*n* = 13; 13.6%), colistin (*n* = 13; 13.6%), fosfomycin (*n* = 13; 13.6%), tigecycline (*n* = 12; 12.6%), ceftolozane/tazobactam (*n* = 9; 9.4%), tobramycin (*n* = 9; 9.4%), cefuroxime-sodium (*n* = 8; 8.4%), moxifloxacin (*n* = 7; 7.3%), norfloxacin (*n* = 5; 5.2%), amikacin (*n* = 5; 5.2%), ceftazidime/avibactam (*n* = 4; 4.2%), cephalotin (*n* = 1; 1.0%), erythromycin (*n* = 1; 1.0%).

Among the 95 isolates, 58 (61.0%) exhibited multidrug resistance (MDR) to antibiotic classes including aminoglycosides, β-lactams, fosfomycin, trimethoprim/sulfamethoxazole, quinolones/fluoroquinolones, piperacillin/tazobactam, nitrofurans, and polymyxins. Phenotypic production of ESBL was identified in 29 (30.5%) isolates, which were recovered from urine (*n* = 10), anal (*n* = 9), blood (*n* = 6), wound (*n* = 2), and sputum (*n* = 2). Many of the ESBL-producing isolates were obtained from elderly individuals (*n* = 12), infants (*n* = 6), adolescents (*n* = 3), middle-aged adults (*n* = 3), adults (*n* = 2), and neonates (*n* = 3). Most of the isolates were recovered from hospitalized patients (*n* = 25). Twenty of the 29 ESBL-producing isolates were also multidrug-resistant.

### 2.2. Whole-Genome Sequencing

Ninety-five *K. pneumoniae* isolates were initially identified in the Diagnostic Laboratory at Farwaniya Hospital using automated identification systems. Of the 95 isolates, 72 were selected for whole-genome sequencing (WGS) based on their antimicrobial susceptibility results, focusing on multidrug-resistant and non-multidrug-resistant isolates. Among the 72 isolates, whole-genome sequence analysis confirmed 58 isolates as *K. pneumoniae*, whereas 14 isolates were identified either as *E. coli* or *Pseudomonas aeruginosa*. Two isolates phenotypically identified as *K. pneumoniae* were identified as *Klebsiella variicola* and *Klebsiella quasipneumoniae* by WGS. One isolate previously identified as *Ewingella americana* by the Microscan automated system was confirmed by WGS as *K. pneumoniae* and was therefore included in the downstream analysis. Among the 58 *K. pneumoniae* isolates, two isolates were excluded because they exhibited high duplication scores (>80%) in BUSCO (Figure S1), which may indicate assembly artifacts or sample contamination. Consequently, a total of 56 *K. pneumoniae* isolates were included in the downstream analysis. All selected isolates showed high BUSCO completeness scores (Figure S1).

The antibiotic susceptibility of the 56 *K. pneumoniae* isolates was as follows: cefuroxime (*n* = 44; 78.5%), ceftazidime (*n* = 44; 78.5%), cefepime (*n* = 39; 69.6%), amoxicillin-clavulanic acid (*n* = 35; 62.5%), ampicillin (*n* = 33; 58.9%), ciprofloxacin (*n* = 33; 58.9%), piperacillin/tazobactam (*n* = 32; 57.1%), trimethoprim/sulfamethoxazole (*n* = 28; 50.0%), meropenem (*n* = 28; 50.0%), cefotaxime (*n* = 26; 46.4%), imipenem (*n* = 24; 42.8%), ertapenem (*n* = 24; 42.8%), ampicillin-sulbactam (*n* = 23; 41.0%), cefazolin (*n* = 21; 37.5%), ceftriaxone (*n* = 21; 37.5%), gentamicin (*n* = 21; 37.5%), nitrofurantoin (*n* = 20; 35.7%), levofloxacin (*n* = 19; 34.6%), aztreonam (*n* = 17; 30.3%), cefoxitin (*n* = 13; 23.2%), colistin (*n* = 11; 19.6%), cefuroxime-axetil (*n* = 10; 17.8%), fosfomycin (*n* = 9; 16.0%), tigecycline (*n* = 9; 16.0%), tobramycin (*n* = 9; 16.0%), moxifloxacin (*n* = 8; 14.2%), ceftolozane/tazobactam (*n* = 7; 12.5%), cefuroxime-sodium (*n* = 6; 10.7%), amikacin (*n* = 5; 8.9%), ceftazidime/avibactam (*n* = 4; 7.1%), norfloxacin (*n* = 3; 5.3%), erythromycin (*n* = 1; 1.7%).

Among the 56 isolates, 47 (83.9%) exhibited multidrug resistance (MDR) to antibiotic classes including aminoglycosides, β-lactams, fosfomycin, trimethoprim/sulfamethoxazole, quinolones/fluoroquinolones, piperacillin/tazobactam, nitrofurans, polymyxins, and macrolides. Phenotypic production of ESBL was identified in 16 (28.5%) isolates, which were recovered from urine (*n* = 7), anal (*n* = 5), blood (*n* = 2), wound (*n* = 1), and sputum (*n* = 1). Many of the ESBL-producing isolates were obtained from elderly individuals (*n* = 6), adolescents (*n* = 3), infants (*n* = 2), middle-aged adults (*n* = 2), adults (*n* = 1), and neonates (*n* = 2). Most of the isolates were recovered from hospitalized patients (*n* = 14). Fifteen of the 16 ESBL-producing isolates were also multidrug-resistant.

The 56 *K. pneumoniae* isolates were further investigated by WGS analysis for the presence of antibiotic resistance genes, virulence-associated genes, mobile genetic elements, and population structure, as given below.

#### 2.2.1. Distribution of Antibiotic Resistance Genes (ARGs) in *K. pneumoniae* Isolates

A complete distribution of antibiotic resistance genes and their variants is presented in Supplementary Table S1. All isolates carried genes encoding resistance to β-lactams (56/56, 100%). The most common ESBL genes were SHV (96.4%), followed by CTX-M-15 (75.0%) and TEM (46.4%) (Table 2 and Table S1). NDM-5 encoding gene was found in 30.4% of the isolates. Forty-four isolates carried genes encoding aminoglycoside resistance (78.5%), with *aac*(*6*′) (55.4%), *aadA* (51.8%), and *aph*(*3*′)*/aph*(*3*″) (46.4%) being the most common genes (Table 2 and Table S1). Two genes encoding resistance to quinolones/fluoroquinolones were identified, with *qnrS* (42.9%) being prevalent among the 56 isolates (Table 2). The sulfonamide resistance gene, *sul*, was detected in 60.7% of the isolates, while the trimethoprim resistance gene *dfrA* was detected in 64.3% of isolates. The rifampicin resistance gene *arr* was found in 10.7%, and the fosfomycin resistance gene *fosA* was detected in all 56 (100%) isolates. Four genes encoding phenicol resistance, i.e., *catB*, *catA*, *catI*, and *cmlA*, were detected in 39.3%, 7.1%, 7.1%, and 3.6% of isolates, respectively. The tetracycline-encoding gene *tet* and its variants were detected in 14.3% of the isolates (Table 2 and Table S1).

#### 2.2.2. Correlation of Antimicrobial Resistance Genotype and Phenotype

A correlation between antimicrobial resistance phenotype and genotype was assessed (Figure 1 and Figure 2). Figure 1 shows the overall agreement between the presence of antibiotic resistance determinants and their corresponding resistance phenotypes across the antibiotic classes tested. In our results, high concordance was observed for cephalosporins (87.5%), penicillins (85.7%), monobactams (77.3%), and carbapenems (57.4%), which is consistent with the widespread distribution of β-lactam resistance genes among the isolates. More than 50% concordance was observed for folate pathway inhibitors (sulfonamides and trimethoprim) and fluoroquinolones. Moderate concordance was noticed for aminoglycosides (44.6%), despite aminoglycoside resistance genes being identified in 78.5% of the isolates. The correlation between Macrolide genotype and phenotype was not assessed because erythromycin susceptibility data were limited to a single isolate.

At the level of individual isolates, the concordance heatmap demonstrated a strong agreement between the presence of resistance genes and resistant phenotypes (gene+/phenotype resistant, green color), particularly for cephalosporins (49/56; 87.5%), penicillins (48/56; 85.7%), and folate pathway inhibitors (28/56; 50%) (Figure 2). In contrast, aminoglycosides (29/56; 51.8%) demonstrated a greater proportion of discrepant, particularly gene+/phenotype susceptible combinations (yellow color), indicating the presence of resistance determinants without corresponding phenotypic resistance (Figure 2). Also, 37.5% and 30.3% of the isolates showed gene+/phenotype susceptible combinations for carbapenems (21/56 isolates) and fluoroquinolones (17/56 isolates), respectively. Nineteen isolates (33.9%) were susceptible to tigecycline (glycylcyclines) and negative for their associated genes (blue color). Among the eight isolates found in the gene negative/phenotype resistant category (red color), seven isolates were found to be associated with tigecycline (glycylcyclines) resistance. Most isolates were not tested for tetracycline and monobactam but carried their corresponding genes (dark gray) (Figure 2).

#### 2.2.3. Distribution and Co-Occurrence of Mobile Genetic Elements with the Antimicrobial Resistance Genes

A total of 10 unique plasmid replicons, 84 insertion sequences (ISs), and four transposons were detected in the 56 *K. pneumoniae* isolates (Table 3). The most common plasmids detected were IncFIB(K) (57.1%) and IncFIB(pKPHS1) (33.9%), which belong to the IncF-family replicons (Table 3). In addition, several small Col-type plasmids, including Col440I, Col440II, ColRNAI, and ColKP3, were detected, with Col440I (48.2%) being the most common. Among the 64 ISs, the predominant were ISSen9 (*n* = 53; 94.6%), IS26 (*n* = 50; 89.3%), and IS903 (*n* = 50; 89.3%). Other common ISs were IS6100 (66.1%), ISEcl10 (66.1%), ISKpn1 (64.3%), ISEcp1(ISEc9) (58.9%), ISKpn26 (55.4%), ISKpn19 (51.8%), ISKpn54 (51.8%), and ISKpn74 (51.8%). The four transposons detected included Tn4352 (1.8%), Tn6196 (1.8%), Tn5403 (1.8%), and Tn4656 (1.8%). The distribution of all plasmids and ISs among the isolates is presented in the Supplementary Table S2.

The analysis of the co-occurrence of antibiotic resistance determinants and mobile genetic elements in the 56 *K. pneumoniae* isolates is presented in Supplementary Table S2. The analysis showed broad associations of plasmid replicons with antimicrobial resistance genes, whereas others were linked to only a limited number of determinants (Table S2). IncFIB(K) revealed the highest number and widest diversity of co-occurring resistance genes, including extended-spectrum β-lactamase genes such as *CTX-M-15*, carbapenemase genes (*NDM-5* and *OXA-1*), aminoglycoside resistance genes (*aac-6*, *aph*(*3*′)*/aph*(*3*″), *aph-*(*6*)) quinolone resistance determinants (*qnrS*), trimethoprim resistance genes (*dfrA*), sulfonamide resistance genes (*sul*1/*sul*2), tetracycline resistance genes (*tetA*), and multiple intrinsic resistance-associated genes (*CRP*, *KpnF*, *mdtB*, *mdtC*, *marA*, *baeR*, *acrB*, *acrD*). Other IncF-family plasmids, including IncFII, IncFIA(HI1), IncR, ColRNAI, and IncHI1B (pNDM-MAR), also demonstrated extensive co-occurrence with numerous antimicrobial resistance genes, although at lower frequencies than IncFIB(K) (Table S2).

For IS elements, the analysis revealed considerable variation in the diversity and frequency of resistance genes associated with different elements (Table S2). IS903 exhibited the broadest range of co-occurring antibiotic resistance determinants, followed by IS26, ISEcp1 (ISEc9), IS6100, ISEcl10, ISKpn1, IS5075, and ISCfr12. These insertion sequences were associated with numerous resistance genes belonging to extended-spectrum β-lactamases (CTX-M-15), carbapenemases (NDM-5, OXA-1, and OXA-181), aminoglycoside-modifying enzyme genes, plasmid-mediated quinolone resistance determinants, fosfomycin resistance genes, trimethoprim resistance genes, sulfonamide resistance genes, and tetracycline resistance genes, in addition to several intrinsic *K. pneumoniae* resistance-associated determinants. In contrast, insertion sequences such as IS30, IS421, IS426, IS630, ISCfr14, ISCfr6, ISEam1, ISEc12, ISEc15, ISEc21, ISEc23, ISEc30, ISEc37, ISEc78, ISEhe2, ISEhe3, ISErsp1, and ISEsa1 were co-occurred with fewer resistance determinants.

The transposon–antimicrobial resistance gene co-occurrence analysis demonstrated that Tn4352 was associated with the greatest diversity and highest frequency of resistance determinants among all transposons identified. Tn4352 co-occurred with numerous resistance genes encoding β-lactamases, aminoglycoside-modifying enzymes, quinolone resistance determinants, fosfomycin resistance genes, and tetracycline resistance genes, in addition to several intrinsic *K. pneumoniae* resistance-associated determinants (Table S2). Tn6196 co-occurred mostly with genes belonging to β-lactam, aminoglycoside, quinolone, and fosfomycin resistance genes, together with intrinsic resistance-associated determinants (Table S2). Tn4656 demonstrated the lowest number and diversity of associated antibiotic resistance genes.

#### 2.2.4. Distribution of Virulence-Associated Genes in *K. pneumoniae*

The distribution of all virulence-associated genes is presented in Supplementary Table S3. Twenty-eight unique virulence-associated genes were detected among the 56 isolates. These genes are involved in iron acquisition, adhesion, and immune evasion (Table 4).

Genes encoding the enterobactin siderophore system (*entA*, *entB*, *fepG*, *and fepC*), adhesion and fimbriae (*ecpA*, *ecpB*, *ecpC*, *ecpD*, *ecpA-E*, *ecpR*), and outer membrane protein A (*ompA*) were detected in more than 90% of the isolates (Table 4). The yersiniabactin siderophore locus, *fyuA*, *irp1*, *irp2*, and *ybt* variant genes were identified in 24 (42.1%) isolates. The aerobactin locus (*iucABCD*, *iutA*) was variably carried in all isolates (Table 4).

#### 2.2.5. Clonal Diversity and Population Structure of *K. pneumoniae* Isolates by Whole-Genome Multilocus Sequence Typing (wgMLST)

The isolates were assigned to 31 sequence types (ST), with 11 and 9 isolates belonging to ST16 and ST147, respectively. Two isolates each belonged to ST37, ST25, ST45, ST348, and ST395. Seventeen sequence types including ST13, ST35, ST231, ST268, ST307, ST461, ST636, ST870, ST873, ST967, ST1083, ST1540, ST1599, ST2096, ST4007, ST7120, and ST7259 were each found in one isolate. Eight isolates were assigned to novel sequence types, including ST9941, ST9946, ST9942, ST9943, ST9944, ST9947, ST9948, and ST9949. The genetic relationships among the 32 STs identified were determined and shown in Figure 3. Sixteen STs were classified into six groups (Table 5). Group 1 consisted of 12 isolates belonging to ST16 (*n* = 11) and a novel ST9942 (*n* = 1). Group 2 consisted of nine ST147 isolates and two novel ST9944 and ST9946. Group 3 comprised of five STs, including ST37, ST35, ST13, ST268, and ST1083, detected in six isolates. Group 4 consisted of ST45 (*n* = 2) and ST1540 (*n* = 1). Group 5 comprised of ST870 (*n* = 1) and ST7120 (*n* = 1), whereas group 6 consisted of ST2096 (*n* = 1) and a novel ST9948 (*n* = 1). The remaining 16 STs were identified as singletons.

The genomic characteristics of the isolates assigned to eight novel STs are presented in Supplementary Table S2. All novel STs were multidrug-resistant isolates except for ST9941 and ST9943, which were non-multidrug-resistant isolates. Isolates belonging to ST9947, ST9948, and ST9949 were β-lactamase producers. ESBL encoding genes, *bla*_CTX-M-15,_ *bla*_SHV-1_, *bla*_SHV-11_, *bla*_SHV-67_, *bla*_SHV-145_, *bla*_SHV-187_, *bla*_NDM-5_, *bla*_TEM-1_, *bla*_TEM-1A_, *bla*_TEM-1B_, *bla*_TEM-105_, *bla*_TEM-150_, and *bla*_OXA-9_ were variably carried by isolates belonging to ST9941, ST9942, ST9944, ST9946, ST9947, ST9948, and ST9949. The *bla*_DHA_ gene was detected in the ST9949 isolate, which was phenotypically resistant to the second- and third- generation cephalosporins and sensitive to the carbapenems (imipenem, meropenem, and ertapenem) and cefepime. The distribution of other AMR genes is shown in Supplementary Table S2. Most isolates carried one or two replicons; however, three isolates belonging to ST9941, ST9942, and ST9949 carried three, seven, and four replicons, respectively. Isolate belonging to ST9942 harbored Col440I, Col440II, ColKP3, IncFIA(HI1), IncFIB(K), IncFII, and IncR, while isolate belonging to ST9949 harbored Col440I, IncFIA(HI1), IncFIB(K), and IncFII. The ST9941 isolate carried IncFIB(K), IncFIB(pKPHS1), and IncFII. Five insertion sequences were detected among isolates belonging to ST9942, ST9944, ST9946, and ST9947. The ST9948 isolate carried four insertion sequences, while the ST994, ST9943, and ST9949 isolates carried three insertion sequences. The most common insertion sequences detected among the isolates were ISSen9 (*n* = 8), IS903 (*n* = 7), and IS26 (*n* = 8). The relatedness of the novel STs is shown in Figure 3. All novel STs were singletons, except for ST9942 and ST9948, which were double-locus variants (DLV) of ST16 and ST2096, respectively. In addition, ST9944 and ST9946 were single-locus variants (SLV) of ST147 (Table 5).

## 3. Discussion

*K. pneumoniae* is a leading cause of healthcare-associated infections and has been recognized by the World Health Organization as a critical-priority pathogen because of its remarkable ability to accumulate antibiotic resistance determinants while causing severe infections in both hospitalized and immunocompromised patients [15,16]. In the present study, whole-genome sequencing was used to determine the antimicrobial resistance determinants, mobile genetic elements, virulence-associated genes, and population structure of 56 *K. pneumoniae* isolates obtained from clinical specimens in Farwaniya Hospital, Kuwait, and to evaluate the relationship between antimicrobial resistance genotype and phenotype. *K. pneumoniae* is one of the leading causes of urinary tract infections and bloodstream infections worldwide [17,18], which is consistent with our findings, in which more than 50% of the isolates were recovered from urine and blood specimens. In addition, 16.8% (*n* = 16) of the isolates were recovered from anal swabs, reflecting gastrointestinal colonization, which represents the primary ecological reservoir for *K. pneumoniae* and a potential source for subsequent endogenous infection and nosocomial transmission, particularly in intensive care units and among elderly, neonatal, and immunocompromised patients [19]. Therefore, surveillance of colonizing isolates provides valuable information regarding the dissemination of multidrug-resistant clones within healthcare settings [19].

The results of the phenotypic susceptibility testing for all 95 *K. pneumoniae* isolates showed high resistance to several β-lactam antibiotics, including cefuroxime, ceftazidime, cefepime, cefotaxime, ceftriaxone, amoxicillin-clavulanic acid, and piperacillin-tazobactam. Resistance to carbapenems, particularly meropenem, imipenem, and ertapenem, was also observed, although at lower frequencies than resistance to extended-spectrum cephalosporins. In addition, 30.5% of the isolates were phenotypically positive for ESBL production, with most ESBL-producing isolates recovered from elderly individuals and infants, who are more susceptible to colonization and infection because of their weak immune systems [20,21]. Although the prevalence of ESBL-producing isolates in the present study was lower than that reported by Jamal et al. [22], who found ESBL production in more than 80% of *K. pneumoniae* isolates in Kuwait, our findings nevertheless indicate the continued circulation of the ESBL-producing *K. pneumoniae* isolates within Kuwait hospitals. Similar findings were also reported in more recent studies from Kuwait, where high resistance rates to penicillins and extended-spectrum cephalosporins, together with lower resistance to carbapenems, were observed [22,23]. Similar to our results, the resistance trends to these antibiotics have also been documented throughout the Gulf Cooperation Council Countries (GCC), including Saudi Arabia, Qatar, and the United Arab Emirates, reflecting the regional expansion of multidrug-resistant *K. pneumoniae* lineages [24,25,26,27].

Whole-genome sequencing demonstrated that these phenotypic resistance patterns for the 56 selected *K. pneumoniae* isolates were caused by diverse acquired antimicrobial resistance genes. In the present study, *bla*_SHV_ was the most prevalent β-lactamase gene family, being detected in 96.4% of the isolates, followed by *bla*_CTX-M-15_ (75.0%), *bla*_OXA_ (64.3%), and *bla*_TEM_ (46.4%). The high prevalence of β-lactamase genes explains the widespread resistance to third- and fourth-generation cephalosporins observed in this study. The predominance of *bla*_SHV_ and *bla*_CTX-M-15_ is consistent with previous reports from Kuwait and neighboring Middle Eastern countries, indicating the persistent circulation of these determinants in the region [22,27,28,29,30,31,32]. Similar findings have also been reported in geographically diverse settings, including Tanzania [33], Portugal [34,35], Romania [36] and Germany [37], demonstrating the widespread distribution of these β-lactamase determinants among *K. pneumoniae* isolates. β-lactamase genes, *bla*_CTX-M-14b_ and *bla*_CTX-M-9,_ were identified only sporadically in this study, suggesting that these variants have not become established in the *K. pneumoniae* population in Kuwait. In addition to their occurrence in human isolates, the *bla*_CTX-M_ gene has also been reported in *K. pneumoniae* isolates from animals [38,39], highlighting the potential role of animal hosts as reservoirs of ESBL genes and underscoring the importance of adopting a One Health approach to address their dissemination.

The carbapenemase gene *bla*_NDM-5_ was detected in 30.4% (*n* = 17) of the *K. pneumoniae* isolates in our study, which is concerning because metallo-β-lactamases hydrolyse nearly all β-lactam antibiotics and are frequently carried together with additional resistance determinants that confer resistance to aminoglycosides, fluoroquinolones, sulfonamides, and trimethoprim [40,41]. The simultaneous detection of *bla*_NDM-5_ with *bla*_CTX-M-15_ and *bla*_OXA_ β-lactamases genes in several isolates in this study further limits treatment options. Similar combinations have been increasingly reported throughout the Middle East and Europe, illustrating the continuing international dissemination of NDM-producing *K. pneumoniae* [42,43,44,45].

The co-occurrence analysis demonstrated that the major β-lactamases, aminoglycoside-modifying enzymes, plasmid-mediated quinolone resistance genes, and folate pathway inhibitor resistance genes were detected in isolates carrying plasmid replicons and insertion sequences. Among the plasmid replicons, IncFIB(K) was the predominant replicon and co-occurred with several antibiotic resistance genes, including *bla*_CTX-M-15_, *bla*_NDM-5_, *bla*_OXA_, *qnrS*, *dfrA*, *sul*, *tetA*, and several intrinsic resistance-associated determinants (Table S2). IncF-family plasmids have been recognized as the major replicons responsible for the international dissemination of *bla*_CTX-M-15_, aminoglycoside resistance genes, and plasmid-mediated quinolone resistance determinants because of their high conjugation efficiency, stability, and capacity to accumulate multiple resistance cassettes [46,47,48]. Another interesting observation is the detection of the IncHI1B(pNDM-MAR) plasmid, which has been recently reported in *bla*_NDM_-carrying *K. pneumoniae* [49,50]. Tn125 is often linked to the dissemination of *bla*_NDM_ [51]; however, it was not found in any of the isolates examined in the present study. Although the detection of IncH1B(pNDM-MAR) and the absence of Tn125 represent noteworthy observations in this study, the cross-sectional nature and single-center focus prevent us from drawing conclusions about temporal changes or the extent to which these components drive *bla*_NDM_ transmission within Kuwait. Among the insertion sequences, ISSen9 was the most prevalent element (94.6%), followed by IS26 and IS903 (both 89.3%). The insertion sequence IS26 detected in this study co-occurred with many clinically important resistance determinants (Table S2). IS26 is a clinically important insertion sequence that has been known to play a role in the dissemination of antimicrobial resistance genes such as *bla*_KPC-2_, *bla*_SHV-5,_ and *bla*_IMP-4_ in multidrug-resistant isolates [52,53].

MLST analysis revealed substantial genetic diversity, with 32 assigned sequence types (STs), including eight novel STs. This heterogeneous population of *K. pneumoniae* isolates indicates that antimicrobial resistance is disseminating across multiple genetic backgrounds rather than through expansion of a single epidemic clone. The identification of six cluster groups together with 16 singleton STs further supports the presence of several independently evolving lineages within the hospital population. In this study, ST16 and ST147 were predominant among the isolates. ST147 is one of the best-characterized multidrug-resistant *K. pneumoniae* lineages and has been reported to be associated with *bla*_CTX-M-15_, *bla*_NDM_, *bla*_OXA-48_-like, aminoglycoside resistance genes, and IncF- and IncHI-type plasmids [47,54,55,56]. Similarly, ST16 has recently emerged as an important carbapenem-resistant lineage in East and Southeast Asia and has been associated with the coexistence of multidrug resistance and enhanced virulence [57,58]. The genomic characteristics of the isolates belonging to the novel STs did not differ substantially from those belonging to the established STs in terms of their antimicrobial resistance gene profiles, virulence genes, and mobile genetic elements (Table S2). These isolates carried multiple β-lactamase genes, including variants belonging to the CTX-M, SHV, NDM, TEM, OXA, and DHA families. Analysis of the MLST allelic profiles, however, demonstrated that some of the novel STs were genetically related to established STs (Table 5). Most of the novel STs were singletons, whereas ST9942 was a DLV of ST16, ST9948 was a DLV of ST2096, and ST9944 and ST9946 were SLVs of ST147. There was no difference in the genetic composition between ST9942, ST9948, ST9944, and ST9946 and their related STs. Although it is difficult to determine whether these novel STs represent the emergence of new lineages in Kuwait, we believe that their presence suggests ongoing genetic diversification within the local *K. pneumoniae* population rather than definitive evidence of newly emerging genetic lineages. These findings highlight the importance of continued genomic surveillance in Kuwait to monitor the evolution and population dynamics of *K. pneumoniae*.

In the present study, the comparison of genomic and phenotypic resistance demonstrated good agreement, particularly for β-lactam antibiotics, which supports the utility of whole-genome sequencing for predicting clinically relevant resistance mechanisms. Strong concordance between the presence of resistance genes and phenotypic resistance was observed for cephalosporins (87.5%), penicillins (85.7%), and monobactams (77.3%), revealing the widespread distribution of β-lactam resistance genes among the 56 isolates. In contrast, aminoglycosides showed lower concordance, which is probably because resistance to these agents is frequently multifactorial and might be influenced by gene expression, efflux pump activity, and chromosomal mutations [59]. Similar discrepancies between genotype and phenotype have been described in several genomic prediction studies, highlighting the continued importance of conventional antimicrobial susceptibility testing alongside genomic surveillance [59,60,61]. While whole-genome sequencing provides a highly informative approach for tracking resistance genes, mobile genetic elements, and epidemic clones, phenotypic susceptibility testing remains indispensable for guiding clinical therapy.

There are limitations to this study, including the results of the phenotypic susceptibility testing, which were inferred from automated identification systems, and the lack of confirmatory disc diffusion testing. Also, the absence of minimum inhibitory concentration (MIC) data limited quantitative evaluation of antimicrobial susceptibility. Another limitation is the single-center design, and the five-month sampling period may limit the generalizability of the findings and may not fully capture the variety of *K. pneumoniae* circulating in Kuwait. In addition, we were unable to fully explore how genomic and AMR properties relate to clinical outcomes due to insufficient clinical metadata.

## 4. Materials and Methods

### 4.1. Collection of K. pneumoniae Isolates

A total of 95 *K. pneumoniae* isolates were collected from clinical specimens from patients in Farwaniya Hospital, Kuwait, during 1 August to 31 December 2024. All isolates were sent to the Microbiology Department, College of Medicine, Kuwait University, and were stored at −80 °C in Luria Broth (LB) (OxoidTM, Hampshire, UK) containing 15% glycerol (Fisher Scientific, Pittsburgh, PA, USA). The study was ethically approved by the Ethical Committee of the Ministry of Health, Kuwait (Research number 1211/2019). The patients/participants (or their legal guardians) provided their written informed consent to participate in this study.

### 4.2. Antimicrobial Susceptibility Testing (AST)

AST was performed using the automated identification systems, VITEK-2 (bioMérieux, Marcy-l’Étoile, France), Phoenix (BD Biosciences, Franklin Lakes, NJ, USA), and Microscan (Beckman Coulter, Brea, CA, USA), according to the manufacturer’s instructions. The antibiotics tested were amikacin, amoxicillin-clavulanic acid, ampicillin, ampicillin-sulbactam, aztreonam, cefazolin, cefepime, cefotaxime, cefoxitin, ceftazidime/clavulanate, ceftazidime, ceftazidime/avibactam, ceftolozane/tazobactam, ceftriaxone, cefuroxime, cefuroxime/axetil, cefuroxime/sodium, cephalothin, ciprofloxacin, colistin, ertapenem, erythromycin, fosfomycin, gentamicin, imipenem, levofloxacin, meropenem, moxifloxacin, norfloxacin, nitrofurantoin, piperacillin/tazobactam, tigecycline, tobramycin, and trimethoprim/sulfamethoxazole.

Due to technical constraints, AST may produce inconsistent susceptibility results for both antimicrobial agents such as colistin and tigecycline, when compared to the standard reference technique. Broth microdilution is the reference technique, particularly for colistin and tigecycline, per CLSI/EUCAST recommendations. In this study, confirmatory broth microdilution was not included, so the susceptibility findings for tigecycline and colistin provided by AST should be interpreted with caution.

### 4.3. DNA Isolation, Library Preparation, and Whole-Genome Sequencing

Genomic DNA (gDNA) was isolated from each *K. pneumoniae* isolate using the Monarch genomic DNA purification Kit (New England Biolabs, Ipswich, MA, USA), as described previously [62]. The isolated gDNA samples were used for the preparation of DNA libraries using the Oxford Nanopore Technologies Ligation library preparation kit, Native Barcoding Kit 96 Vl4 (Oxford, UK) as specified by the kit manufacturer. The prepared libraries were sequenced using the R10.4.1 FLO-MIN 114-flow cell on a MinION MklC device from Oxford Nanopore Technologies (Oxford, UK), as described previously [62].

### 4.4. Bioinformatics for Data Analyses

Several bioinformatics software, pipelines, and tools were used for data analyses. These included MinKNOW software for basecalling and demultiplexing. The MinKNOW built-in Dorado (2.0.1 version) basecaller converts MinION fast5 files into fastq files during sequencing. A default q-score threshold of 7 was used to separate reads into fastq pass (>7) and fastq fail (≤7) (https://nanoporetech.com/software/other/dorado) (accessed on 4 November 2025). Zcat (version 1.14) was used for combining multiple fastq files into a single fastq file (https://www.biostars.org/p/334887/) (accessed on 4 November 2025). Nanoplot (version 1.46.1) was employed to filter the reads to include only those with a quality score >10 (https://github.com/wdecoster/NanoPlot) (accessed on 4 November 2025) [63]. Porechop (version 0.2.4) was used for trimming barcodes and adaptors (https://github.com/rrwick/Porechop) (accessed on 4 November 2025). Flye (version 2.9.5-b1801) was used for de novo assembly (https://github.com/fenderglass/Flye) (accessed on 4 November 2025). QUAST (version 5.3.0) was used to evaluate the quality of all the corrected assemblies (https://github.com/ablab/quast) (accessed on 9 November 2025). Benchmarking Universal Single-Copy Orthologs (BUSCO) (version 6.0.0) was employed to assess the completeness of the assembled genome with the bacteria_odb10 lineage (https://github.com/metashot/busco) (accessed on 9 November 2025) [64]. ABRicate (version l.0.1) with six databases; Virulence Factors Database (VFDB), the comprehensive antibiotic resistance database (CARD), Antibiotic Resistance Gene-ANNOTation (ARG-ANNOT), (ResFinder) Resistance Finder, NCBI (National Center for Biotechnology Information) AMRFinderPlus for identifying antimicrobial resistance genes, virulence factors, and stress response genes in bacterial genomic data, (PlasmidFinder) for identifying plasmid replicons in the bacterial genome sequences. For species identification, 16S rRNA gene sequences were compared against the NCBI database. A minimum spanning tree was generated using the PHYLOViZ (version 2.0) online (https://online.phyloviz.net/index) (accessed on 16 April 2025).

## 5. Conclusions

To the best of our knowledge, this is the first comprehensive genomic study of *K. pneumoniae* in Kuwait to characterize 56 isolates using whole-genome sequencing. This study provided a detailed overview of the genomic characteristics, antimicrobial resistance determinants, and population structure of *K. pneumoniae* circulating in Kuwait. The findings revealed a high prevalence of the ESBL-associated genes *bla*_SHV_, *bla*_CTX-M-15_, *bla*_OXA_, and *bla*_TEM_, together with a high prevalence of specific plasmid replicons, particularly IncFIB(K), highlighting the potential contribution of mobile genetic elements to the dissemination of antimicrobial resistance. Most isolates belonged to the two internationally recognized high-risk clones, ST16 and ST147, while eight novel sequence types (STs) were also identified, further demonstrating the genetic diversity of the *K. pneumoniae* population in Kuwait. Overall, this study contributes to the current understanding of the genomic characteristics of *K. pneumoniae* in Kuwait and highlights the importance of continuous genomic surveillance. Such surveillance can provide valuable insights to support clinicians and infection control teams in optimizing patient treatment, clinical management, and infection prevention strategies.

## Figures and Tables

**Figure 1:**
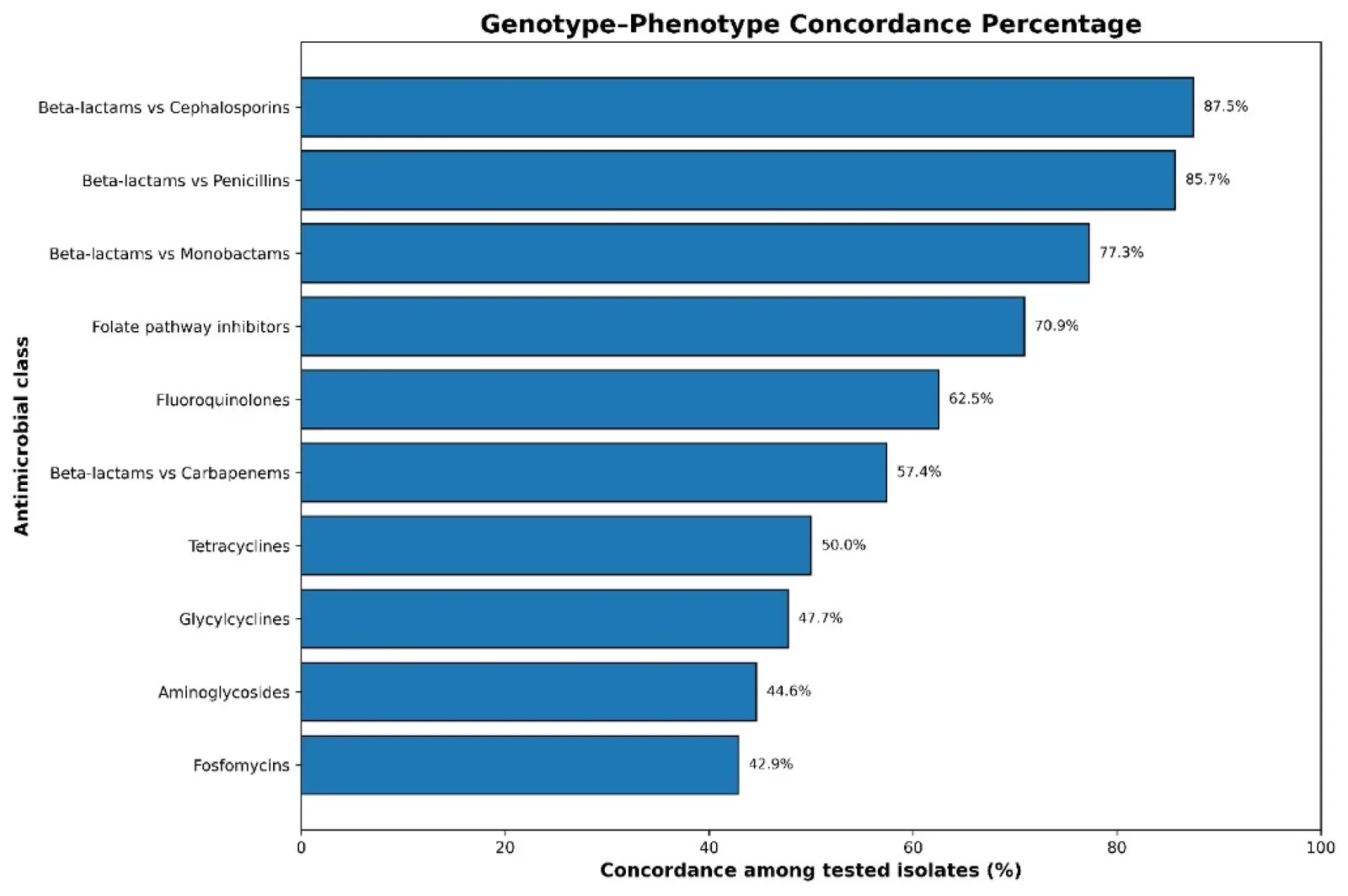
Genotype–phenotype concordance for antimicrobial resistance among *K. pneumoniae* isolates. Bars represent the percentage of phenotypically resistant isolates carrying the corresponding resistance determinants for each antimicrobial class.

**Figure 2:**
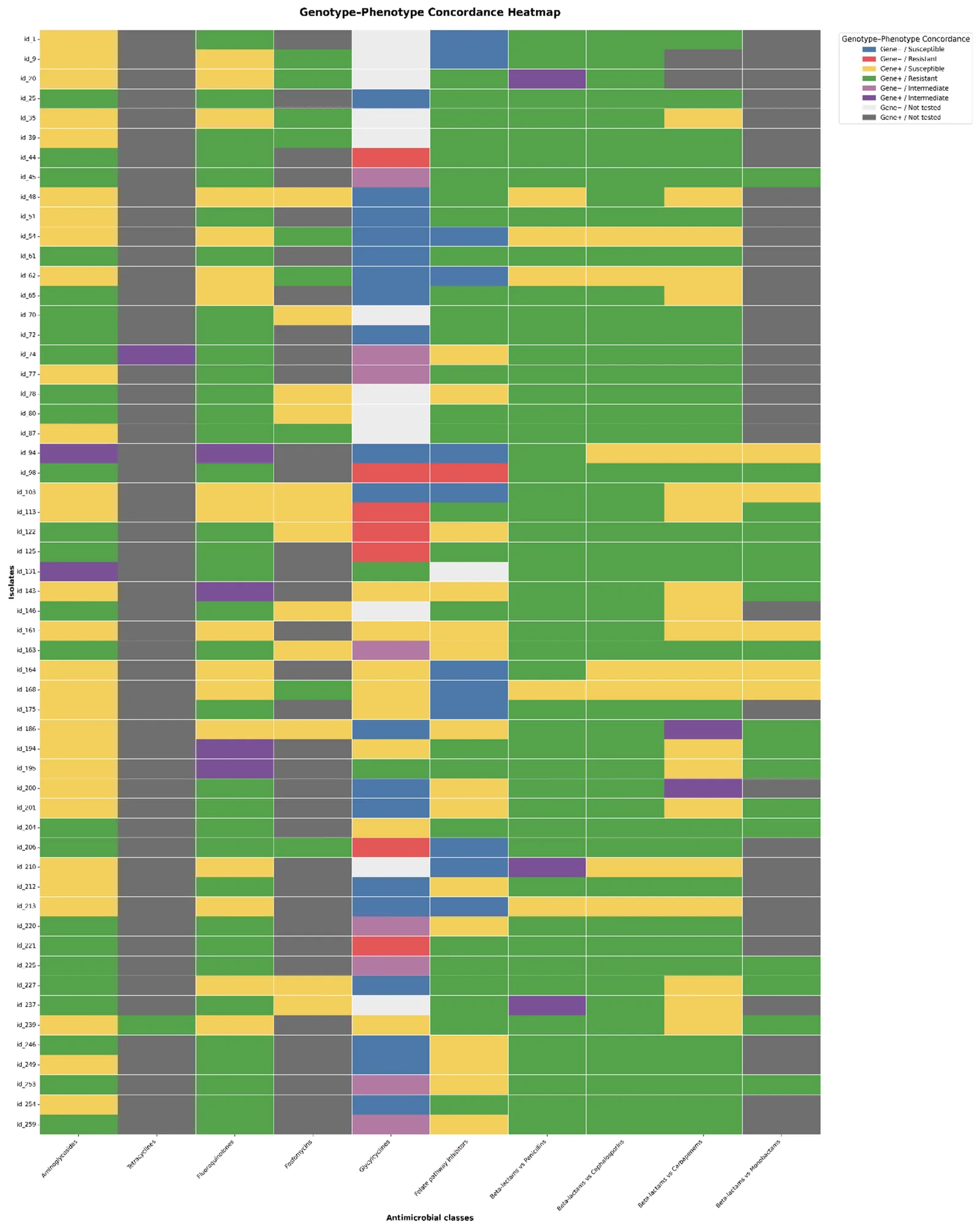
Heatmap illustrates the concordance between phenotypic antimicrobial susceptibility testing and resistance determinants identified by whole-genome sequencing among *K. pneumoniae* isolates. Rows represent all 56 isolates, and columns represent antimicrobial classes. Colors represent the genotype–phenotype concordance categories: concordant resistant (green), concordant susceptible (blue), genotype-positive/phenotype-susceptible (yellow), genotype-negative/phenotype-resistant (red), genotype-positive/intermediate phenotype (dark purple), genotype-negative/intermediate phenotype (light purple), genotype-negative/phenotype not tested (light gray), and genotype-positive/phenotype not tested (dark gray).

**Figure 3:**
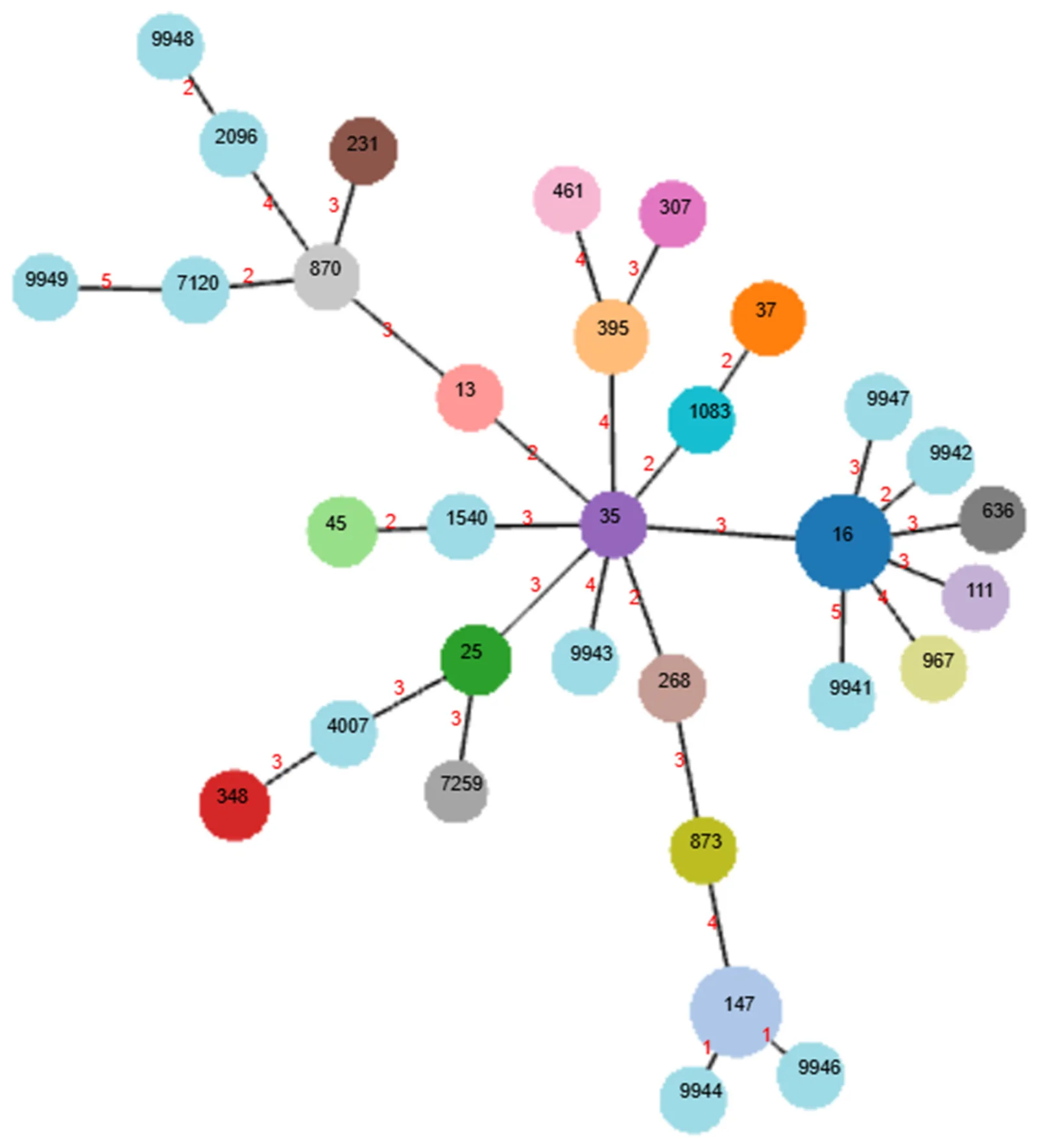
Minimum spanning tree generated using PHYLOViZ from the seven-locus multilocus sequence typing (MLST) allelic profiles. Node size is proportional to the number of isolates assigned to each ST. Connecting lines indicate the shortest allelic relationships between STs, and the red numbers on the connecting lines represent the number of allelic differences between STs.

**Table 1:** Patients’ demographic data.

Patient Age	No.
Neonates: Birth to 28 days	5
Infants: 29 days to 1 year	10
Children: 1 year to 9 years	0
Adolescents: 10 years to 19 years	5
Adults: 20 to 39 years	15
Middle-aged adults: 40 to 59 years	21
Older adults/elderly: 60 years and above	39
**Total**	95

**Table 2:** Distribution of major antibiotic resistance genes among *K. pneumoniae* isolates.

Antimicrobial Class (n)	AMR Gene	No. of Isolates	%
β-lactams (56)	Penicillin-binding protein	55	98.2
	*ampH*	55	98.2
	*bla*_SHV_	54	96.4
	*bla*_CTX-M-15_	42	75.0
	*bla*_CTX-M-14b_	1	1.8
	*bla*_CTX-M-9_	1	1.8
	*bla*_OXA_	36	64.3
	*bla*_TEM_	26	46.4
	*bla*_NDM-5_	17	30.4
	*bla*_DHA_	3	5.4
	*LEN*	1	1.8
	*LEN16*	1	1.8
	*OKP-B*	1	1.8
Aminoglycosides (44)	*aac*(*6*′)	31	55.4
	*aadA*	29	51.8
	*aph*(*3*′)*/aph*(*3*″)	26	46.4
	*aac*(*3*)	18	32.1
	*ant*(*3*″)	17	30.4
	*aph*(*6*)	15	26.8
	*Str*	15	26.8
	*aphA6*	13	23.2
	*rmtB*	6	10.7
	*rmtF*	1	1.8
Quinolones/fluoroquinolones (27)	*qnrS*	24	42.9
	*qnrB*	3	5.4
Macrolides (26)	*Mph*	25	44.6
	*Erm*	3	5.4
	*Ere*	2	3.6
Phenicols (27)	*catB*	22	39.3
	*catA*	4	7.1
	*catI*	4	7.1
	*cmlA*	2	3.6
Fosfomycin (56)	*fosA*	56	100
Sulfonamides (34)	*sul*	34	60.7
Trimethoprim (36)	*dfrA*	36	64.3
Tetracyclines (8)	*tet*	8	14.3
Rifampicin (6)	*arr*	6	10.7
Streptothricin (2)	*sat*	2	3.6
Mupirocin (1)	*mupA*	1	1.8

**Table 3:** Major mobile genetic elements associated with antimicrobial resistance among *K. pneumoniae* isolates.

Mobile Genetic Element	No. of Isolates	%
**Plasmid replicons**		
IncFIB(K)	32	57.1
Col440I	27	48.2
IncFIB(pKPHS1)	19	33.9
ColRNAI	15	26.8
Col440II	14	25
IncFIA(HI1)	15	26.8
ColKP3	12	21.4
IncFIB(Mar)	12	21.4
IncHI1B(pNDM-MAR)	12	21.4
IncFII	12	21.4
**Insertion sequences**		
ISSen9	53	94.6
IS26	50	89.3
IS903	50	89.3
IS6100	37	66.1
ISEcl10	37	66.1
ISKpn1	36	64.3
ISEcp1 (ISEc9)	33	58.9
ISKpn26	31	55.4
ISKpn19	29	51.8
ISKpn54	29	51.8
ISKpn74	29	51.8
IS5075	27	48.2
ISCfr12	23	41.1
ISEcl1	23	41.1
ISEc33	22	39.3
ISEc38	19	33.9
ISKpn21	18	32.1
ISKpn60	16	28.6
ISKqu3	16	28.6
ISEc27	14	25
ISEsa2	14	25
ISKpn38	14	25
ISKpn65	14	25
ISAba125	13	23.2
ISKox3	13	23.2
ISRor6	13	23.2
ISAs26	12	21.4
ISKpn41	12	21.4
ISSty2	12	21.4
ISKpn24	11	19.6
ISKpn28	11	19.6
ISEch12	9	16.1
IS102	8	14.3
ISKox1	8	14.3
ISSen4	8	14.3
ISEc52	7	12.5
ISKpn14	7	12.5
ISEc36	6	10.7
ISEc23	5	8.9
ISKpn25	5	8.9
ISVsa3	5	8.9
ISEhe3	4	7.1
ISKpn2	4	7.1
ISKpn34	4	7.1
IS5	3	5.4
ISCfr14	3	5.4
ISEam1	3	5.4
ISKpn43	3	5.4
ISPlge3	3	5.4
ISSba14	3	5.4
ISSen3	3	5.4
IS630	2	3.6
ISCfr6	2	3.6
ISEc15	2	3.6
ISEhe2	2	3.6
ISKpn18	2	3.6
ISKpn42	2	3.6
ISKpn49	2	3.6
ISKpn63	2	3.6
ISPan1	2	3.6
ISRaq1	2	3.6
ISRor3	2	3.6
ISSpr2	2	3.6
ISYps7	2	3.6
**Integrative conjugative elements**		
ICEKpnHS11286-1	1	1.8
ICEEcoED1a-1	1	1.8
**Transposons**		
Tn4352	1	1.8
Tn6196	1	1.8
Tn5403	1	1.8
Tn4656	1	1.8

**Table 4:** Distribution of major virulence genes among the *K. pneumoniae* isolates.

Virulence Category	Gene	No. of Isolates (%)
Adhesion and fimbriae	*yagV/ecpE*	56 (98.2)
Adhesion and fimbriae	*yagW/ecpD*	56 (98.2)
Adhesion and fimbriae	*yagX/ecpC*	56 (98.2)
Adhesion and fimbriae	*yagY/ecpB*	56 (98.2)
Adhesion and fimbriae	*yagZ/ecpA*	56 (98.2)
Adhesion and fimbriae	*ykgK/ecpR*	56 (98.2)
Iron acquisition systems	*entA*	53 (93.0)
Iron acquisition systems	*entB*	56 (98.2)
Iron acquisition systems	*espX4*	1 (1.8)
Iron acquisition systems	*fepC*	56 (98.2)
Iron acquisition systems	*fepG*	13 (22.8)
Iron acquisition systems	*fyuA*	24 (42.1)
Iron acquisition systems	*irp1*	24 (42.1)
Iron acquisition systems	*irp2*	24 (42.1)
Iron acquisition systems	*iucA*	15 (26.3)
Iron acquisition systems	*iucB*	15 (26.3)
Iron acquisition systems	*iucC*	14 (24.6)
Iron acquisition systems	*iucD*	1 (1.8)
Iron acquisition systems	*iutA*	14 (24.6)
Iron acquisition systems	*ybtA*	24 (42.1)
Iron acquisition systems	*ybtE*	24 (42.1)
Iron acquisition systems	*ybtP*	24 (42.1)
Iron acquisition systems	*ybtQ*	24 (42.1)
Iron acquisition systems	*ybtS*	24 (42.1)
Iron acquisition systems	*ybtT*	24 (42.1)
Iron acquisition systems	*ybtU*	24 (42.1)
Iron acquisition systems	*ybtX*	24 (42.1)
Outer membrane and immune evasion	*ompA*	56 (98.2)

**Table 5:** The genetic relatedness of the sequence types (STs) identified in *K. pneumoniae* isolates.

Groups	ST	Frequency	SLV	DLV	Allelic Profile						
					*gapA*	*infB*	*mdh*	*pgi*	*phoE*	*rpoB*	*tonB*
Group 1	16	11			2	1	2	1	4	4	4
	9942	1		1	2	1	2	652	4	4	77
Group 2	147	9			3	4	6	1	7	4	38
	9944	1	1		580	4	6	1	7	4	38
	9946	1	1		3	413	6	1	7	4	38
Group 3	35	1			2	1	2	1	10	1	19
	37	3		1	2	9	2	1	13	1	16
	13	1		1	2	3	1	1	10	1	19
	268	1		1	2	1	2	1	7	1	81
	1083	1		1	2	1	2	1	13	1	23
Group 4	45	2			2	1	1	6	7	1	12
	1540	1		1	2	1	1	6	10	1	62
Group 5	870	1			2	6	1	1	147	1	31
	7120	1		1	2	6	1	1	11	1	15
Group 6	2096	1			1	6	1	1	1	46	1
	9948	1		1	1	6	1	653	1	46	1445

Abbreviations: SLV, single-locus variant; DLV, double-locus variant.

## Data Availability

All data are available in this paper and the Supplementary Tables S1–S3. The whole-genome sequencing data reported in this study have been deposited in the NCBI GeneBank database under BioProject accession number PRJNA1499723.

## Supplementary Materials

The following supporting information can be downloaded at: https://www.mdpi.com/article/10.3390/antibiotics15100960/s1, Figure S1: BUSCO assessment for all *K. pneumoniae* isolates; Table S1: AMR genes; Table S2: co-occurance_of_AMR_genes_and_MGE_56_isolates; Table S3: Virulence associated genes for the 56 isolates.
